# Cumulative (Dis)Advantage and the Matthew Effect in Life-Course Analysis

**DOI:** 10.1371/journal.pone.0142447

**Published:** 2015-11-25

**Authors:** Miia Bask, Mikael Bask

**Affiliations:** 1 Norwegian Social Research (NOVA), Oslo and Akershus University College of Applied Sciences, Oslo, Norway; 2 Department of Economics, Uppsala University, Uppsala, Sweden; Universiteit Gent, BELGIUM

## Abstract

To foster a deeper understanding of the mechanisms behind inequality in society, it is crucial to work with well-defined concepts associated with such mechanisms. The aim of this paper is to define cumulative (dis)advantage and the Matthew effect. We argue that cumulative (dis)advantage is an intra-individual micro-level phenomenon, that the Matthew effect is an inter-individual macro-level phenomenon and that an appropriate measure of the Matthew effect focuses on the mechanism or dynamic process that generates inequality. The Matthew mechanism is, therefore, a better name for the phenomenon, where we provide a novel measure of the mechanism, including a proof-of-principle analysis using disposable personal income data. Finally, because socio-economic theory should be able to explain cumulative (dis)advantage and the Matthew mechanism when they are detected in data, we discuss the types of models that may explain the phenomena. We argue that interactions-based models in the literature traditions of analytical sociology and statistical mechanics serve this purpose.

## Introduction

The overall objective of this paper is to promote a deeper understanding of the mechanisms behind inequality in society by discussing two concepts associated with such mechanisms: *cumulative advantage* or *disadvantage* and the *Matthew effect*. We argue that cumulative (dis)advantage is an intra-individual micro-level phenomenon, that the Matthew effect is an inter-individual macro-level phenomenon and that this difference in phenomena has consequences for the modeling of socio-economic processes that may explain cumulative (dis)advantage and the Matthew effect when they are detected in data. We also provide a novel measure of the Matthew effect that focuses on a property of the dynamic process that generates inequality, including a proof-of-principle analysis using disposable personal income data that shows how this measure can be estimated from data.

A popular model in life-course research that has achieved widespread acceptance in the literature that attempts to explain inequality in society is the cumulative advantage model proposed by Crystal and Shea [1]. This model focuses on how inequality can be magnified over a life course because people accumulate different amounts of advantages and disadvantages over time: *“those who are initially advantaged […] are more likely to receive a good education*, *leading to good jobs*, *leading to better health and better pension coverage*, *leading to higher savings and better postretirement benefit income”* (p. 437 in [1]). Similarly, those who are initially disadvantaged are less likely to receive a good education, leading to poor jobs, leading to worse health and worse pension coverage, leading to lower savings and worse postretirement benefit income. Thus, intra-cohort inequality is magnified over a life course because people accumulate different amounts of advantages and disadvantages over time.

One concept that is closely related to the idea of cumulative (dis)advantage is the Matthew effect. This term is derived from the Gospel of Matthew, in which Jesus says, *“[f]or unto every one that hath shall be given*, *and he shall have abundance*: *but from him that hath not shall be taken away even that which he hath”* (Matthew 25:29). Although the Matthew effect is referenced in life-course research (see [2] for an early example), its use is more common in the sociology of science due to Merton’s [3–4] observation that better-known scientists tend to receive more academic recognition than lesser-known scientists for similar achievements. Consequently, better-known scientists attract more resources at the expense of lesser-known scientists, which widens the gap between the two groups’ resources and achievements.

Clearly, cumulative (dis)advantage and the Matthew effect are fruitful concepts in life-course research. However, as DiPrete and Eirich [5] noted in their review of different cumulative (dis)advantage processes and how they may lead to increased inequality, if further progress in research is to be made, there is a need for more explicit attention to methodological issues in the application of different concepts associated with inequality-generating processes. The aim of the present paper, therefore, is to define cumulative (dis)advantage and the Matthew effect. Moreover, because socio-economic theory should be able to explain cumulative (dis)advantage and the Matthew effect when they are detected in data, we argue that a heterogeneous agent model is needed to explain the phenomena.

We argue that an appropriate measure of the Matthew effect focuses on the *mechanism* or dynamic process that generates inequality rather than on the outcome of the process per se. Thus, to better understand inequality-generating mechanisms and how they affect, for example, individuals’ life courses—and to avoid unintentionally overlooking important aspects of the dynamics—one should measure the Matthew effect and not, so to speak, the effect of the Matthew effect. Thus, we argue that the *Matthew mechanism* is a better name for the phenomenon. In any event, our measure of the Matthew mechanism, which we present below (in the section Defining the Matthew mechanism), coincides with the Mertonian understanding of this concept (referred to here as the Matthew doctrine):

“Taken out of its spiritual context and placed in a wholly secular context, the Matthew doctrine would seem to hold that the posited process must result in a boundlessly growing inequality of wealth, however wealth is construed in any sphere of human activity. Conceived of as a locally ongoing process and not as a single event, the practice of giving unto everyone that hath much while taking from everyone that hath little will lead to the rich getting forever richer while the poor become poorer. Increasingly absolute and not only relative deprivation would be the continuing order of the day. But as we know, things are not as simple as all that. After all, the extrapolation of local exponentials is notoriously misleading”

(pp. 609–610 in [4]).

R.K. Merton also wrote the following regarding the Matthew effect, or the Matthew mechanism:

“[T]he Matthew effect is the accruing of large increments of peer recognition to scientists of great repute for particular contributions in contrast to the minimizing or withholding of such recognition for scientists who have not yet made their mark. The biblical parable generates a corresponding sociological parable”

(p. 609 in [4]).

Three things are worth noting in these quotations. First, the Matthew mechanism is a process that results in increased inequality. Second, this process is ongoing and, therefore, dynamic. Third, R.K. Merton correctly claimed that such a dynamic process may lead to boundlessly increasing inequality. However, as we discuss below (in the section Defining the Matthew mechanism), some dynamic processes are bounded in the sense that no one becomes infinitely rich; however, these processes are still *“capable of magnifying small differences over time and [make] it difficult for an individual or group that is behind at a point in time […] to catch up”* (p. 272 in [5]), which is one of the characteristics of an inequality-generating process as identified by DiPrete and Eirich [5].

R.K. Merton compared the *“recognition to scientists of great repute”* to *“scientists who have not yet made their mark”* in the second quotation, thereby making an inter-individual comparison of scientists’ levels of recognition. Similarly, in the first quotation, he made an inter-individual comparison of wealth. As we discuss below (in the section Distinguishing between cumulative (dis)advantage and the Matthew mechanism), we also understand the Matthew mechanism as an inter-individual phenomenon. Moreover, we understand cumulative (dis)advantage as an intra-individual phenomenon. Accordingly, those who become *“forever richer”* in R.K. Merton’s first quotation experience cumulative advantage, and those who *“become poorer”* experience cumulative disadvantage.

The rest of this paper is organized as follows. In the section Distinguishing between cumulative (dis)advantage and the Matthew mechanism, we make a distinction between cumulative (dis)advantage and the Matthew mechanism in the context of people’s life courses and inequality in society. In the section Defining the Matthew mechanism, we properly define the Matthew mechanism. In the section A proof-of-principle analysis, we provide a proof-of-principle analysis using disposable personal income data. In the section What is the value added by measuring *λ*?, we discuss the value added of our proposed measure of the Matthew mechanism. The section Explaining cumulative (dis)advantage and the Matthew mechanism concludes the paper with a discussion of the types of models that may explain cumulative (dis)advantage and the Matthew mechanism because socio-economic theory should be able to explain the phenomena when they are detected in data.

## Distinguishing between Cumulative (Dis)Advantage and the Matthew Mechanism

Consider a large population in which two individuals are named Adam and Eve, and assume that the socio-economic status of Adam is described by the state variable, or *n*-tuple, $S_{t}^{Adam}\in {\mathbb{R}}^{n}$ at time *t*. Thus, the socio-economic status of Adam is described with the help of *n* variables, which may include educational level, income level, and occupational status:

$$S_{t}^{Adam}=[education_{t}^{Adam},income_{t}^{Adam},occupation_{t}^{Adam}].$$ (1)

It is reasonable to assume that Eve’s socio-economic status, $S_{t}^{Eve}\in {\mathbb{R}}^{n}$, can be described similarly:

$$S_{t}^{Eve}=\left[education_{t}^{Eve},income_{t}^{Eve},occupation_{t}^{Eve}\right].$$ (2)

The ordering of the variables in the *n*-tuples is usually important. However, because the exact ordering of the variables does not matter for the measure of the Matthew mechanism that we present below (in the section Defining the Matthew mechanism), we do not elaborate on this issue here. Instead, we proceed to the following question: is there some natural measure that represents the socio-economic inequality that exists between Adam and Eve and how this inequality changes over time?

Let us first define the distance between Adam’s and Eve’s socio-economic statuses. Naturally, at time *t* = 0, the distance between their socio-economic statuses is defined as

$$d_{0}^{Adam,Eve}\;\equiv \;\|S_{0}^{Adam}\;-\;S_{0}^{Eve}\|.$$ (3)

Let us thereafter calculate the distance between Adam’s and Eve’s socio-economic statuses at times *t* = 1, *t* = 2, and so on, up to and including time *t* = *t*_*max*_, which gives us the time series ${\left\{d_{t}^{Adam,Eve}\in \mathbb{R}\right\}}_{t=0}^{t_{max}}$. Note that to be able to calculate the distance in Eq (3), we need to know all of the values of the *n* variables that define Adam’s socio-economic status as well as all of the values of the *n* variables that define Eve’s socio-economic status. However, this is *not* necessary when examining whether the Matthew mechanism is present in the process that generates Adam’s and Eve’s socio-economic statuses (see the section A proof-of-principle analysis).

**Fig 1:**
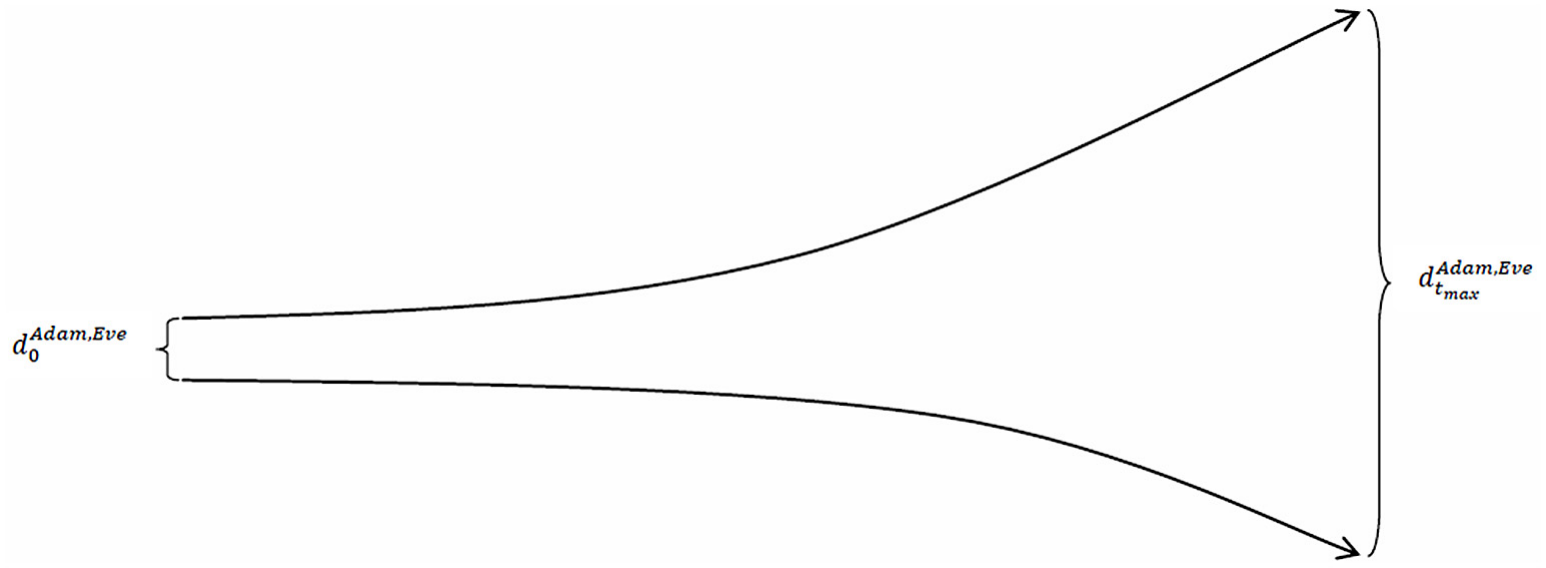
The distance between Adam’s and Eve’s socio-economic statuses increases over time.

**Fig 2:**
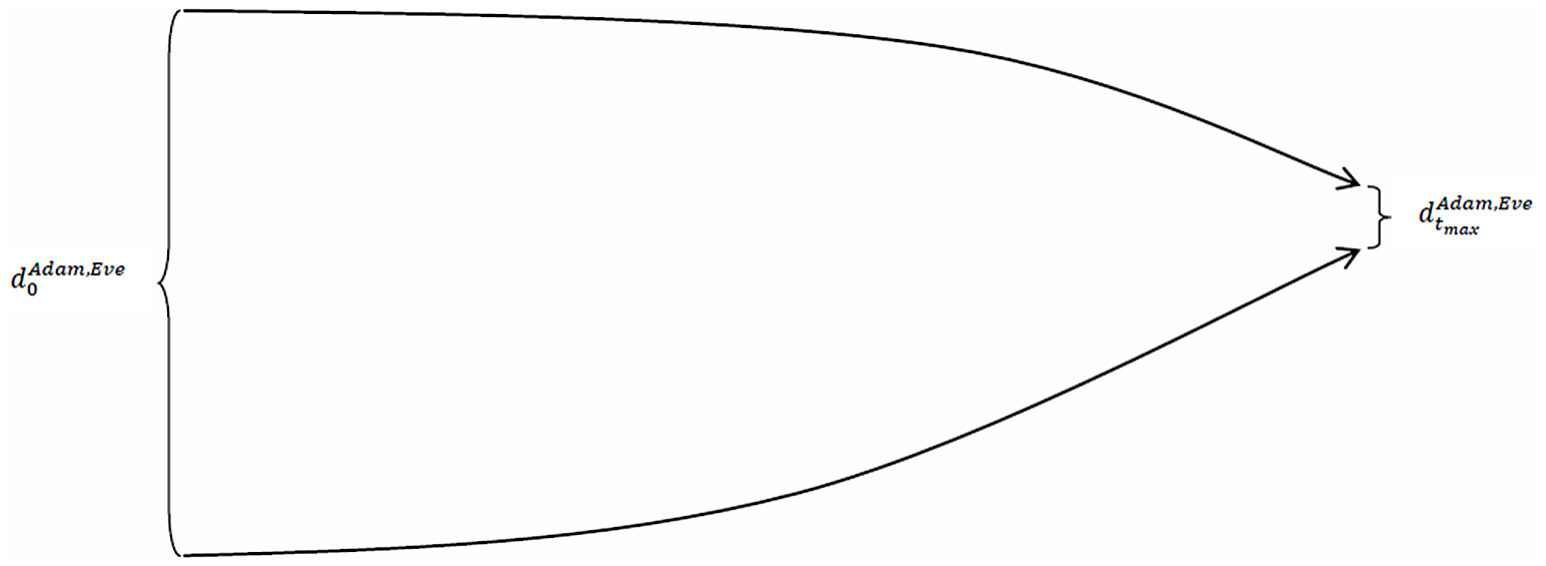
The distance between Adam’s and Eve’s socio-economic statuses decreases over time.

If the trend in the aforementioned time series sloped upward, then the trajectories of Adam’s and Eve’s socio-economic statuses would diverge over time, which means that we would have an inter-individual divergence of trajectories. This pattern in the time paths of individuals’ trajectories is typically interpreted as the Matthew effect. This effect, however, can be described in a more sophisticated way if we anticipate how it is defined below (in the section Defining the Matthew mechanism): an inter-individual divergence of individuals’ trajectories is a sign that the *mechanism* generating the trajectories is the *Matthew mechanism*. Conversely, if the trend in the same time series sloped downward, then the trajectories of Adam’s and Eve’s socio-economic statuses would converge over time, which means that we would have an inter-individual convergence of trajectories. Figs 1 and 2 illustrate these cases.

**Fig 3:**
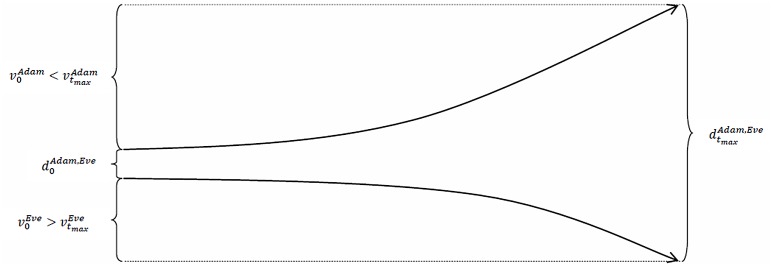
The distance between Adam’s and Eve’s socio-economic statuses increases over time when Adam experiences a cumulative advantage and Eve experiences a cumulative disadvantage.

**Fig 4:**
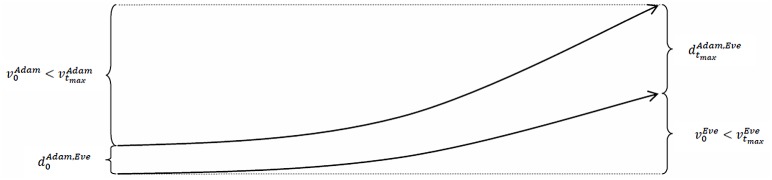
The distance between Adam’s and Eve’s socio-economic statuses increases over time even when both Adam and Eve experience a cumulative advantage.

**Fig 5:**
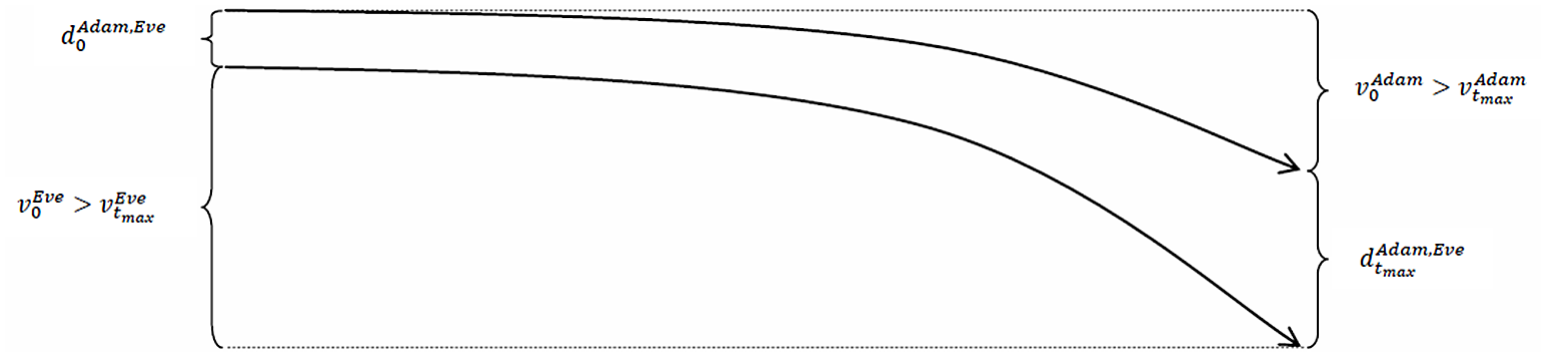
The distance between Adam’s and Eve’s socio-economic statuses increases over time even when both Adam and Eve experience a cumulative disadvantage.

If we start with the Matthew mechanism, the divergence of the trajectories of Adam’s and Eve’s socio-economic statuses can have one of three possible causes. First, Adam experiences a *cumulative advantage* because the time series of the values of his socio-economic status, ${\left\{v_{t}^{Adam}\in \mathbb{R}\right\}}_{t=0}^{t_{max}}$, where

$$v_{t}^{Adam}\;\equiv \;d_{t}^{Adam,0}\;\equiv \;\|S_{t}^{Adam}-[0,\ldots ,0]\|,$$ (4)

slopes upward. In contrast, Eve’s time series of the values of her socio-economic status, ${\left\{v_{t}^{Eve}\right\}}_{t=0}^{t_{max}}$ (defined in a similar way as in Eq (4)), slopes downward, which means that she experiences a *cumulative disadvantage* (or that Adam experiences a cumulative disadvantage and Eve experiences a cumulative advantage). Second, both individuals experience a cumulative advantage, but Adam’s (or Eve’s) time series of the values of his (or her) socio-economic status is more strongly upward-sloping than Eve’s (or Adam’s) time series of values. Third, both individuals experience a cumulative disadvantage, but Adam’s (or Eve’s) time series of the values of his (or her) socio-economic status is less strongly downward-sloping than Eve’s (or Adam’s) time series of values. Figs 3, 4 and 5 illustrate these cases.

The convergence of the trajectories of Adam’s and Eve’s socio-economic statuses can also have one of three possible causes: (i) Adam (or Eve) experiences a cumulative advantage, whereas Eve (or Adam) experiences a cumulative disadvantage; (ii) both of them experience a cumulative advantage, but Adam’s (or Eve’s) time series of the values of his (or her) socio-economic status is more strongly upward-sloping than Eve’s (or Adam’s) time series of values; or (iii) both of them experience a cumulative disadvantage, but Adam’s (or Eve’s) time series of the values of his (or her) socio-economic status is less strongly downward-sloping than Eve’s (or Adam’s) time series of values. Thus, we see the same cases as above when we observed the effect of the Matthew mechanism. The key difference here, of course, is whether Adam or Eve had the better socio-economic status at time *t* = 0 (i.e., if $v_{0}^{Adam}>v_{0}^{Eve}$, or if $v_{0}^{Adam}<v_{0}^{Eve}$).

The simple point we would like to make here is that there is no one-to-one correspondence between intra-individual change in socio-economic status (which is the result of either cumulative advantage or cumulative disadvantage), on the one hand, and inter-individual convergence or divergence of the trajectories of individuals’ socio-economic statuses (which, in the case of divergence, is the result of the Matthew mechanism), on the other. There is an obvious explanation for the lack of such a clear-cut relationship: the *intra*-individual change in socio-economic status is a *micro*-level phenomenon, whereas the *inter*-individual change in socio-economic status is a *macro*-level phenomenon. This difference in phenomena has consequences for the modeling of socio-economic processes that may explain cumulative (dis)advantage and the Matthew mechanism (cf. [6] and Coleman’s boat in [7]).

How do we define the Matthew mechanism if we introduce Cain and Abel to our story? One route is to compare the trajectories of individuals’ socio-economic statuses within each pair of trajectories in the population by looking at the trends in the following six time series: ${\left\{d_{t}^{Adam,Eve}\right\}}_{t=0}^{t_{max}}$, ${\left\{d_{t}^{Adam,Cain}\right\}}_{t=0}^{t_{max}}$, ${\left\{d_{t}^{Adam,Abel}\right\}}_{t=0}^{t_{max}}$, ${\left\{d_{t}^{Eve,Cain}\right\}}_{t=0}^{t_{max}}$, ${\left\{d_{t}^{Eve,Abel}\right\}}_{t=0}^{t_{max}}$, and ${\left\{d_{t}^{Cain,Abel}\right\}}_{t=0}^{t_{max}}$. Specifically, if the trends in all of these time series slope upward, we can identify the Matthew mechanism because all of the individuals’ trajectories diverge from one another. However, such a definition of the Matthew mechanism would be too restrictive.

For example, there is a situation in which all of the time series listed above have upward-sloping trends except for one time series: ${\left\{d_{t}^{Cain,Abel}\right\}}_{t=0}^{t_{max}}$. The reason for the downward-sloping trend in this time series may be that Cain killed Abel at time *t* = *t*_0_ and that, as a result, Abel’s socio-economic status abruptly dropped to an *n*-tuple with zeros, $S_{t_{0}}^{Abel}=\left[0,\ldots ,0\right]$. Cain’s socio-economic status also decreased rapidly and the explanation for the convergence of their trajectories is that Abel’s initial socio-economic status was higher than Cain’s (because God accepted Abel’s sacrifice but rejected Cain’s; of course, all individuals appearing in this paper are fictitious, and any resemblance to real persons, living or dead, is purely coincidental).

Although the example is simplistic, it illustrates that we do not observe the Matthew mechanism if we adopt the definition that *all* individuals’ trajectories must diverge from one another to have such a mechanism. This idea is not satisfactory. A better definition of the Matthew mechanism may be that after taking the average of the slopes of all of the time series showing how the distance between two individuals’ socio-economic statuses evolves over time, the average slope should be positive if the Matthew mechanism is involved. However, even though this definition represents an improvement over the former definition, it still suffers from drawbacks.

The first drawback relates to what DiPrete and Eirich [5] argued is a well-defined inequality-generating process. Namely, because it is difficult for individuals who are behind at a particular instant in time to catch up with the others, an inequality-generating process should be capable of magnifying small differences over time; thus, we should restrict our attention to the pairs of trajectories of individuals’ socio-economic statuses that were initially close to each other. The second drawback is that we may not only be interested in comparing trajectories that were close at the *same* time; we might also wish to compare trajectories that were close at *different* times (i.e., *ε* is small):

$$d_{t_{0},t_{1}}^{Adam,Eve}\;\equiv \;\|S_{t_{0}}^{Adam}\;-\;S_{t_{1}}^{Eve}\|\;<\;\varepsilon .$$ (5)

One might also argue that the comparison in Eq (5) should be further restricted to individuals who belong to the same birth cohort because two individuals of different ages with similar socio-economic statuses are not comparable; one has had a longer period of time to achieve his or her socio-economic status than the other.

However, there is a more fundamental problem with the definitions above; they are all based on individuals’ socio-economic trajectories. As a result, the definitions neglect variables that affect or are affected by individuals’ socio-economic statuses without defining the statuses themselves. Health status provides an example: Adam’s health status might affect both his own and Eve’s socio-economic statuses if they are cohabiting (because poor health status reduces the chance of earning a high income), and it might also be the case that Adam’s socio-economic status affects both his and Eve’s health statuses (because low income reduces the chance of receiving good health care). Thus, to develop a more profound understanding of the causes of inequality in society, we must find a new measure of the Matthew effect, or the Matthew mechanism, that addresses the aforementioned problems in a more careful and insightful way.

## Defining the Matthew Mechanism

Here, we present a measure of the Matthew mechanism that is similar in spirit to the measure discussed above (in the section Distinguishing between cumulative (dis)advantage and the Matthew mechanism) but that circumvents the aforementioned problems by shifting the focus from individuals’ trajectories to the *dynamic process* that generates these trajectories. This shift in focus also enables us to develop a deeper understanding of the causes of inequality in society because our new measure not only takes into account how an individual’s socio-economic status interacts with other individuals’ socio-economic statuses but also how it interacts with individuals’ health statuses and other relevant variables. In fact, to measure the Matthew mechanism, we do not have to keep track of all of the variables that affect, or are affected by, individuals’ socio-economic statuses (see the section A proof-of-principle analysis).

Specifically, the dynamic process that generates the life courses of all individuals in a given population is denoted by $f\;:{\mathbb{R}}^{n_{f}}\to {\mathbb{R}}^{n_{f}}$ and expresses how the life-course state ${𝒮}_{t}\in {\mathbb{R}}^{n_{f}}$ of the process evolves over time:

$${𝒮}_{t+1}=f\left({𝒮}_{t}\right).$$ (6)

Let us first define the life-course state ${𝒮}_{t}$ before we discuss the properties of the dynamic process *f*(·) and present our measure of the Matthew mechanism.

Recall that we described the socio-economic statuses of Adam and Eve using *n* variables. In fact, each individual in the population has a socio-economic status that can be described by *n* variables. The life-course state ${𝒮}_{t}$ of the dynamic process *f*(·) consists of *n* socio-economic variables as well but also includes variables that affect or are affected by individuals’ socio-economic statuses without defining the statuses themselves. More concretely, we interpret a life course as *“a sequence of socially defined events and roles that the individual enacts over time”* (p. 22 in [8]), which means that an individual’s socio-economic status is a subset of the same individual’s life-course state. Bear in mind our discussion of Adam’s and Eve’s health statuses and how they may affect or be affected by their socio-economic statuses.

With regard to the properties of the dynamic process *f*(·), because *f*(·) generates all individuals’ life courses in a given population, the process does not end when an individual in the population, such as Abel, dies. Therefore, it is necessary to assume that *f*(·) is bounded, which is an often overlooked assumption in the somewhat sparse literature on inequality-generating processes. Note that it is not necessary to assume that *f*(·) belongs to a certain function class for our measure of the Matthew mechanism to exist, even though a specific socio-economic theory, presented in mathematical form, would imply that *f*(·) belongs to a certain function class.

The dynamic process *f*(·) amplifies the distance between two life-course states, ${𝒮}_{t}$ and ${𝒮}_{t}^{\prime }$, where the initial distance $d_{0}\equiv \left\|{𝒮}_{0}-{𝒮}_{0}^{\prime }\right\|\;<\varepsilon$ between the states is short (i.e., *ε* is small):

$${𝒮}_{t}-{𝒮}_{t}^{\prime }=f^{t}({𝒮}_{0})-f^{t}({𝒮}_{0}^{\prime })\;≅\;Df^{t}({𝒮}_{0})({𝒮}_{0}-{𝒮}_{0}^{\prime }),$$ (7)

where

$$Df^{t}({𝒮}_{0})=Df({𝒮}_{t-1})Df({𝒮}_{t-2})\;\cdots Df({𝒮}_{0}).$$ (8)

We are interested in how the distance $d_{t}\equiv \;\left\|{𝒮}_{t}-{𝒮}_{t}^{\prime }\right\|\;\in \mathbb{R}$ between the life-course states ${𝒮}_{t}$ and ${𝒮}_{t}^{\prime }$ of the dynamic process *f*(·) is amplified when time approaches infinity (i.e., $\underset{t\to \infty }{\text{lim}}d_{t}$). For this aim, we measure the Lyapunov characteristic exponent, λ∈R, which is defined by the following limit:

$$\lambda \;\equiv \;\underset{t\to \infty }{\text{lim}}\frac{{\text{log}}_{e}\left|Df^{t}({𝒮}_{0})\right|}{t}\;=\;\underset{t\to \infty }{\text{lim}}\frac{1}{t}\Sigma_{k=0}^{t-1}{\text{log}}_{e}\left|Df({𝒮}_{k})\right|.$$ (9)

Under suitable technical conditions, the limit in Eq (9) exists and is independent of life-course state ${𝒮}_{0}$ [9–10].

In particular, if *λ* > 0, the dynamic process *f*(·) has the property that any two life-course trajectories with arbitrarily close, but not identical, life-course states will diverge from each other at an exponential rate even if they remain within a bounded space. Specifically, if the life-course state of *f*(·) is, for instance, ${𝒮}_{0}^{Adam}$, we should observe the following series of states: ${𝒮}_{1}^{Adam}$, ${𝒮}_{2}^{Adam}$, and so on. Alternatively, if the life-course state of *f*(·) is, for example, ${𝒮}_{0}^{Eve}$, we should observe the following series of states: ${𝒮}_{1}^{Eve}$, ${𝒮}_{2}^{Eve}$, and so on. Then, if *λ* > 0 and if Adam’s and Eve’s initial life-course states are close, then their life-course trajectories, ${\left\{{𝒮}_{t}^{Adam}\right\}}_{t=0}^{t_{max}}$ and ${\left\{{𝒮}_{t}^{Eve}\right\}}_{t=0}^{t_{max}}$, will diverge from each other. In other words, *λ* > 0 encapsulates what DiPrete and Eirich [5] argued is a well-defined inequality-generating process because it is *“capable of magnifying small differences over time and makes it difficult for an individual or group that is behind at a point in time […] to catch up”* (p. 272 in [5]).

Furthermore, every dynamic process *f*(·) has a Lyapunov characteristic exponent, *λ*. However, *λ* < 0 cannot be associated with the Matthew mechanism because it is associated with a point in phase space; thus, there is no systematic divergence of the life-course trajectories. Furthermore, *λ* = 0 cannot be associated with the Matthew mechanism because it is associated with life-course trajectories with self-sustained and (quasi-) periodic oscillations in phase space, meaning that there is no systematic divergence of the trajectories in this case either. Thus, *λ* > 0 is a necessary condition for the Matthew mechanism to occur. The question is, therefore, whether *λ* > 0 is also a sufficient condition. Because we do not place any restriction on the speed of the divergence of the life-course trajectories, we do not place any numerical restriction on *λ* other than that it should be positive; this means that *λ* > 0 is a necessary and sufficient condition for the Matthew mechanism to hold.

Note that when two life-course states, for example, ${𝒮}_{t}^{Adam}$ and ${𝒮}_{t}^{Eve}$, are close, the corresponding socio-economic statuses, $S_{t}^{Adam}$ and $S_{t}^{Eve}$, are also close. Thus, if the Matthew mechanism is present in the dynamic process *f*(·) that generates Adam’s and Eve’s life courses, then not only will their life-course trajectories diverge over time but so will their socio-economic trajectories. However, when two socio-economic statuses, $S_{t}^{Adam}$ and $S_{t}^{Eve}$, are close, this does not necessarily mean that the corresponding life-course states, ${𝒮}_{t}^{Adam}$ and ${𝒮}_{t}^{Eve}$, are close. This is because the socio-economic statuses are proper subsets of the life-course states. Consequently, we cannot expect that Adam’s and Eve’s socio-economic trajectories will diverge over time in this case, even if *f*(·) is characterized by the Matthew mechanism.

However, recall that we interpret an individual’s life course as *“a sequence of socially defined events and roles that the individual enacts over time”* (p. 22 in [8]). If we borrow Mayer’s [11] words as a complement to this understanding, *“[w]ith the term life course sociologists denote the sequence of activities or states and events in various life domains spanning from birth to death”* (p. 163). Thus, if we neglect the possibility that variables other than socio-economic variables also affect or are affected by individuals’ socio-economic statuses, we could incorrectly conclude that the Matthew mechanism is not in play when individuals’ socio-economic trajectories do not diverge from each other in longitudinal studies. This conclusion emphasizes the fact that an appropriate measure of the Matthew mechanism should focus on the dynamic process that generates inequality rather than on the outcome of the process per se.

## A Proof-of-Principle Analysis

Although it is natural to assume that one must know the actual form of the dynamic process *f*(·) to be able estimate our measure of the Matthew mechanism, *λ*, this assumption is not valid. Instead, using the celebrated embedding theorem of Takens [12], it is possible to reconstruct the dynamics using only a scalar time series and then to estimate *λ* of the reconstructed process (see [13] for a geometric illustration of the embedding theorem). Moreover, because asymptotic theory is available for statistical inferences, a scalar time series of a variable generated by *f*(·) is sufficient to conclude whether the Matthew mechanism is present in this process. The software NETLE 4.1 may be used for this task. NETLE 4.1 was developed by R. Gençay, C.-M. Kuan, and T. Liu. This software can be downloaded from http://tliu.iweb.bsu.edu/download/index.html.

Associate the unknown dynamic process *f*(·) with the observer function $g\;:{\mathbb{R}}^{n_{f}}\to \mathbb{R}$ that generates

$$s_{t}=g\left({𝒮}_{t}\right)+\varepsilon_{t},$$ (10)

where *s*_*t*_ is the reconstruction variable and *ε*_*t*_ is the measurement error. Hence, the time series ${\left\{s_{t}\right\}}_{t=0}^{t_{max}}$ is observed. In our proof-of-principle analysis, the reconstruction variable is disposable personal income for Average Joe, $s_{t}=income_{t}^{Average\;Joe}$, for the period 1947 Q1 through 2015 Q1. Specifically, the reconstruction variable is the log-difference of the quarterly U.S. per capita (hence, the name Average Joe) disposable personal income in chained 2009 U.S. Dollars.

The *t*_*max*_ + 1 observations in the time series contain information on unobserved state variables that can be utilized to define a state in the present time. For this reason, let

$$T={\left(T_{0},T_{1},\ldots ,T_{n_{T}-1}\right)}^{\prime }$$ (11)

be the reconstructed trajectory that describes how the reconstructed state $T_{t}\in {\mathbb{R}}^{n_{h}}$ evolves over time; additionally, let $n_{T}$ be the number of states in the reconstructed trajectory. Moreover, the reconstructed state at time *t* is

$$T_{t}=\left\{s_{t},s_{t+1},\ldots ,s_{t+n_{h}-1}\right\},$$ (12)

where *n*_*h*_ is the embedding dimension. Thus, T is an $n_{T}\times n_{h}$ matrix, and the constants $n_{T}$, *n*_*h*_, and *t*_*max*_ are related as $n_{T}=t_{max}-n_{h}+2$.

Takens [12] proved that the function

$$\Phi \left({𝒮}_{t}\right)=\left\{g\left(f^{0}\left({𝒮}_{t}\right)\right),g\left(f^{1}\left({𝒮}_{t}\right)\right),\ldots ,g\left(f^{n_{h}-1}\left({𝒮}_{t}\right)\right)\right\},$$ (13)

which maps the *n*_*f*_-dimensional unobserved state ${𝒮}_{t}$ onto the *n*_*h*_-dimensional reconstructed state $T_{t}$, is an embedding when *n*_*h*_ > 2*n*_*f*_. Thus, the function $\Phi \;:{\mathbb{R}}^{n_{f}}\to {\mathbb{R}}^{n_{h}}$ preserves topological information about the unknown dynamic process *f*(·), such as the Lyapunov characteristic exponent. In particular, the function induces another function, $h\;:{\mathbb{R}}^{n_{h}}\to {\mathbb{R}}^{n_{h}}$, on the reconstructed trajectory,

$$T_{t+1}=h\left(T_{t}\right),$$ (14)

which is topologically conjugate to *f*(·):

$$h^{j}\;=\;\Phi \;\circ \;f^{j}\circ \Phi^{-1}(T_{t}).$$ (15)

*h*(·) is, therefore, a reconstructed dynamic process that has the same Lyapunov characteristic exponent as the unknown dynamic process *f*(·).

Shintani and Linton [14] derived the asymptotic distribution of a neural network estimator of the Lyapunov characteristic exponent, *λ*, which is our measure of the Matthew mechanism. Specifically, the neural networks are estimated by the method of nonlinear least squares [15], where the Lyapunov characteristic exponent is calculated from the derivative matrices of the estimated neural networks [16]. Using NETLE 4.1, we estimated *λ* making use of 3, 4, 5, 6, 7, 8, 9, 10, 11 and 12 inputs to the neural network, respectively, where the number of hidden units ran, in each case, from 2 to 12 units. Thus, we estimated 110 neural networks. We then selected the estimate of the Lyapunov characteristic exponent, $\hat{\lambda }$, associated with the neural network that minimized the Schwarz Information Criterion. We found that the Matthew mechanism was not present in the dynamic process that generated disposal personal income for Average Joe since $\hat{\lambda }\;=\;-0.40$ (*p* value = 0.04) (see S1 File for data and software).

## What Is the Value Added by Measuring *λ*?

What is the value added by using *λ* as a measure of the Matthew mechanism? Is this a better measure of inequality than, for example, the Gini index (see [17] for an overview of different inequality measures)? It is not a better measure simply because *λ* is not exactly an inequality measure, even though the Matthew mechanism is closely related to inequality, as noted in the introductory section and in the quotations from Merton [4]. An inequality measure such as the Gini index (or any Lorentz-curve based measure, including the recently proposed *k* index by [18]) measures the degree of, for example, socio-economic inequality between individuals at a certain point in time, whereas *λ* measures how the degree of inequality changes over time between individuals with similar life-course states. Thus, *λ* > 0, which indicates the presence of the Matthew mechanism, can be associated with both a low Gini index and a high Gini index. Therefore, *λ* is a complement to an inequality measure such as the Gini index.

It is also important not to confuse inequality with unfairness. This is because a fairness measure is a value-based measure that depends upon the prevailing opinion of what is considered to be a fair distribution of resources; this is not the case with an inequality measure such as the Gini index. Moreover, the fairness of a specific distribution of resources can be viewed differently depending on the circumstances in which the resources have been achieved. Take the income distribution as an example; does it only depend on differences in occupation and skill, or is it also affected by race and sex? In other words, because *λ* measures how the degree of inequality changes over time between individuals with similar life-course states, it is not a fairness measure.

If *λ* is neither an inequality measure nor a fairness measure, is it a measure of the Matthew mechanism? To address this question, we refer to Merton’s [4] description of the typical characteristics of the Matthew effect, or the Matthew mechanism. First, the Matthew mechanism is a process that results in inequality. Second, the Matthew mechanism is an ongoing process and is, therefore, dynamic. Third, the Matthew mechanism is a non-linear dynamic process because it is bounded but still capable of magnifying small differences between individuals’ life-course states over time. Thus, *λ* > 0 is an appropriate measure of the Matthew mechanism because any two life-course trajectories with arbitrarily close but non-identical life-course states will diverge from one another.

However, Merton’s [3–4] definition should not be the only benchmark with which to characterize the Matthew mechanism. We complement the descriptions of the Matthew mechanism by Merton [3–4] with those of Dannefer [2], who provided an early example of the Matthew mechanism (or the Matthew effect, in his terminology) in life-course research, and DiPrete and Eirich [5], who called for more explicit attention to methodological issues in the application of different concepts associated with inequality-generating processes. Dannefer [2] noted that *“several types of social processes may tend to generate a Matthew effect within each cohort over its collective life course”* and that *“[t]he Matthew effect is consistent with the frequently observed trend of increasing intracohort […] inequality with advancing age”* (pp. 216–217). Thus, he interpreted the Matthew effect, or the Matthew mechanism, as an intra-cohort phenomenon, or, equivalently, as an inter-individual phenomenon, as we do.

DiPrete and Eirich [5] identified three characteristics of the Matthew mechanism (or cumulative advantage, in their terminology). First, the Matthew mechanism is a *“mechanism for inequality across any temporal process […] in which a favorable relative position becomes a resource that produces further relative gains”* (p. 271 in [5]). Second, the Matthew mechanism *“becomes part of an explanation for growing inequality when current levels of accumulation have a direct causal relationship on future levels of accumulation”* (p. 272 in [5]). Third, the Matthew mechanism *“is capable of magnifying small differences over time and makes it difficult for an individual or group that is behind at a point in time […] to catch up”* (p. 272 in [5]). Thus, the characteristics listed here are, more or less, the same as those emphasized by Merton [3–4]. Based on the works of Dannefer [2], DiPrete and Eirich [5], and Merton [3–4], we conclude that the Matthew mechanism is in play when *λ* > 0.

Although the discussion in this paper is centered on people’s life courses and inequality in society, our measure of the Matthew mechanism may find empirical applications in other areas of the social sciences (see [19] for one such example).

## Explaining Cumulative (Dis)Advantage and the Matthew Mechanism

If cumulative (dis)advantage and the Matthew mechanism are detected in data, socio-economic theory must be able to explain the phenomena; otherwise, the theory would not explain some of the important properties for which it is supposed to account. Rigney [20] wrote the following regarding the Matthew mechanism (or the Matthew effect, in his terminology):

“The study of Matthew effects […] explores the mechanisms or processes through which inequalities, once they come into existence, become self-perpetuating and self-amplifying in the absence of intervention, widening the gap between those who have more and those who have less. No theory of stratification is complete without attention to such processes”

(p. 2 in [20]).

Because we have argued that the outcome of a cumulative (dis)advantage process is an intra-individual micro-level phenomenon, whereas the outcome of a Matthew mechanism process is an inter-individual macro-level phenomenon, a model that explains cumulative (dis)advantage and the Matthew mechanism must be a heterogeneous agent model. Compare with Allison et al. [6] who wrote that a *“model of cumulative advantage does not imply increasing inequality*. *When the model is modified to allow for heterogeneity in the rate of cumulative advantage*, *however*, *increasing inequality is implied”* (p. 615). Note that they interpreted cumulative (dis)advantage as an intra-individual phenomenon, as we do.

One problem with the cumulative advantage model in life-course research, as it is portrayed in the introductory section, is that the model does not explain *why* people accumulate different amounts of advantages and disadvantages over time. The reason is that the model only provides a so-called statistical explanation of why we observe increased intra-cohort inequality over time. In other words, the explanation identifies variables such as education level, health status, and pension coverage, which seem to be important for the probability of the observed phenomenon to occur. A better explanation would be a mechanism-based explanation because such an explanation is based on the actors in society and their (inter-)actions and is, therefore, able to explain *why* a better educated person may receive a better job or *why* a better job may lead to better health.

Finding mechanism-based explanations of social phenomena is part of the core of analytical sociology (see [21] and selected contributions in the edited volumes by [22–23] on analytical sociology). A broad group of heterogeneous agent models in this literature tradition are interactions-based models. The unifying characteristic of this group of models is that they are used to study (inter-)actions between agents in a wide range of contexts; Schelling’s [24] racial segregation model is an early example of such a model. The literature on interactions-based models is too vast to review here (see instead [25] for a review of this literature). We are not aware of any work that explicitly addresses and explains, in a unified theoretical framework, the micro- and macro-level (life-course) phenomena described in this paper. However, interesting research in this direction is presented by Manzo and Baldassarri [26].

There is currently a large body of literature that argues that several social phenomena are characterized by emergent behavior, meaning that the behavior of a social system does not depend on its individual parts but on the relationships between the different parts. Consequently, the behavior of a single agent in an emergent social system cannot predict the behavior of the whole system. (One can mention in this context that our proposed measure of the Matthew mechanism, *λ*, has also been used a predictability measure for a dynamic process; see [10].) This situation has led researchers to use tools and insights from statistical mechanics when studying social phenomena (see [27] for a review of this literature). In fact, the marriage of agent-based modeling and the use of statistical mechanics when analyzing social phenomena has resulted in a new branch of the social sciences, sociophysics (see [28] for an introductory text to this subject).

We believe that the development of interactions-based models in the literature tradition of analytical sociology that aim to explain cumulative (dis)advantage and the Matthew mechanism in a unified theoretical framework should go hand in hand with the use of tools and insights gained in statistical mechanics. The reason is that interactions-based models, or agent-based models, together with the toolbox of statistical physics have shown promise in shedding light on different collective phenomena [29]. A deeper understanding of the mechanisms behind people’s life courses might be more challenging to achieve than an understanding of most other social phenomena because it involves insights from such different disciplines as economics, public health, social psychology, and sociology. Nevertheless, this work should be a subject of further research.

## Supporting Information

S1 File The folder “Data and Software.zip” contains all the 53 files needed to replicate the empirical analysis in this paper. (ZIP)
